# The Association between Prenatal Yoga and the Administration of Ritodrine Hydrochloride during Pregnancy: An Adjunct Study of the Japan Environment and Children’s Study

**DOI:** 10.1371/journal.pone.0158155

**Published:** 2016-06-27

**Authors:** Yasuyuki Kawanishi, Yasuaki Saijo, Eiji Yoshioka, Yoshihiko Nakagi, Takahiko Yoshida, Toshinobu Miyamoto, Kazuo Sengoku, Yoshiya Ito, Chihiro Miyashita, Atsuko Araki, Reiko Kishi

**Affiliations:** 1 Department of Health Science, Asahikawa Medical University, Asahikawa, Hokkaido, Japan; 2 Department of Obstetrics and Gynecology, Asahikawa Medical University, Asahikawa, Hokkaido, Japan; 3 Faculty of Nursing, Japanese Red Cross Hokkaido College of Nursing, Kitami, Hokkaido, Japan; 4 Center for Environmental and Health Sciences, Hokkaido University, Sapporo, Hokkaido, Japan; Chiba University Center for Forensic Mental Health, JAPAN

## Abstract

**Introduction:**

While the beneficial effects of prenatal yoga have been reported in recent years, little is known about its effectiveness in pregnant Japanese women. Despite several adverse effects, ritodrine hydrochloride is frequently prescribed to suppress preterm labor in Japan, and its usage may therefore indicate cases of preterm labor. This study aimed to clarify the association between prenatal yoga and ritodrine hydrochloride use during pregnancy.

**Methods:**

An observational study was conducted as an adjunct study by the Hokkaido unit of the Japan Environment and Children’s Study. Information on prenatal yoga practice was collected using a self-questionnaire between March 21, 2012, and July 7, 2015, targeting women who had recently delivered. Ritodrine hydrochloride use was identified from medical records. A total of 2,692 women were analyzed using logistic regression models that adjusted for possible confounders.

**Results:**

There were 567 (21.1%) women who practiced prenatal yoga, which was associated with a lower risk of ritodrine hydrochloride use (adjusted odds ratio [OR] 0.77; 95% CI 0.61–0.98). This was especially evident in women with a total practice duration that exceeded 900 minutes throughout their pregnancy (adjusted OR 0.54; 95% CI 0.38–0.76). A sensitivity analysis that excluded patients with threatened abortion during the study period produced similar results.

**Conclusions:**

Prenatal yoga was associated with a lower risk of ritodrine hydrochloride use, particularly in women with more than 900 minutes of practice time over the course of their pregnancy. Prenatal yoga may be a beneficial option for pregnant women in the selection of alternative therapies.

## Introduction

Yoga is a mental and physical practice with origins in ancient Indian philosophy [1]. Previous research based on randomized controlled trials (RCTs) has suggested that yoga may reduce low back pain [2], improve vitality in breast cancer patients, and reduce inflammatory cytokines [3]. In recent years, prenatal yoga has also been reported to reduce psychiatric stress and anxiety [4,5] low back pain [6], pregnancy complications in high-risk patients (such as those with obesity or advanced age) [7], and the duration of labor in healthy pregnant women [8]. The beneficial effects of prenatal yoga have been previously summarized elsewhere [9].

Two RCTs have also investigated the effects of yoga on neonatal prematurity: a study from the US reported significantly longer mean gestational durations in women who practiced yoga (yoga group, mean (SD), 38.6 (1.9) weeks; non-yoga group, 36.7 (2.6) weeks) [10]. Similarly, a study from India observed a significantly lower prevalence of preterm births (yoga group, 20.7%; control group, 45.7%) among women with high-risk factors such as history of obstetric complications, obesity, or advanced age [7]. However, studies have yet to examine the effects of prenatal yoga on prematurity or tocolytic drug use in pregnant Japanese women [9], and there is currently little evidence to support the practice of prenatal yoga in Japan.

Preterm birth is a major contributing factor to perinatal morbidity and mortality [11,12]. Drugs such as β2-agonists are frequently administered throughout the world (particularly in developing countries) to treat preterm labor [13], which if left untreated can lead to spontaneous preterm birth. Despite evidence supporting the effectiveness of betamimetics in reducing the number of women in preterm labor who give birth within 48 hours, these drugs have not been observed to actually reduce the incidence of preterm births [13]. Due to the relatively high risk of adverse effects and the lack of effectiveness in preventing preterm births, the usage of betamimetics tends to be avoided, particularly among developed countries [13,14].

The proportion of preterm births in Japan is among the lowest in the world [15–17]. The key drug for treating preterm labor in Japan is ritodrine hydrochloride, which is a β-agonist [18–20]. While the Japanese Ministry of Health, Labour and Welfare approved another tocolytic agent (magnesium sulfate) for the treatment of preterm labor in 2008, its use is restricted to cases that are unresponsive to ritodrine hydrochloride. As a result, ritodrine hydrochloride remains the de facto first-line drug for preterm labor in Japan [19]. Despite its widespread use, ritodrine hydrochloride places a substantial burden on the cardiovascular system [21] and may also result in other adverse effects[22–25], indicating that it should only be administered when necessary. Hence, identifying alternative options that can contribute to a decrease in ritodrine hydrochloride use would be beneficial for pregnant women. In this study, we investigated the use of prenatal yoga as a possible alternative to reduce preterm labor in pregnant women.

This observational study aimed to clarify the association between prenatal yoga and the use of ritodrine hydrochloride during pregnancy.

## Methods

### Study Participants

The Japan Environment and Children’s Study (JECS) [26] is an ongoing large-scale prospective birth cohort study, and its protocol has been previously described in detail [27]. Women are recruited in the early stages of pregnancy, and a total of 103,106 women throughout Japan participated in this study between January 1, 2011 and March 31, 2014 [28]. The Hokkaido unit is one of 15 regional centers of the JECS, and had recruited 8,362 pregnant women at the time of the study. The Hokkaido unit administrates participants from 3 areas: Sapporo area (Kita-ward and Toyohira-ward of Sapporo city; total population, 497,000), Asahikawa area (Asahikawa city; population, 346,000) and Kitami area (Kitami city, Okedo town, Kunneppu town, Tsubetsu town, and Bihoro town; total population, 155,000).

This study was conducted as an adjunct study outlined in the JECS protocol paper [27]. The study protocol was approved by the Japanese Ministry of the Environment. This adjunct study on the effects of prenatal yoga was performed on JECS Hokkaido unit participants using self-questionnaires. Participants provided written informed consent before inclusion in the study. Explanatory materials and self-questionnaires regarding prenatal yoga were sent to eligible study participants after delivery, and the questionnaires were collected between March 21, 2012 and July 7, 2015.

**Fig 1:**
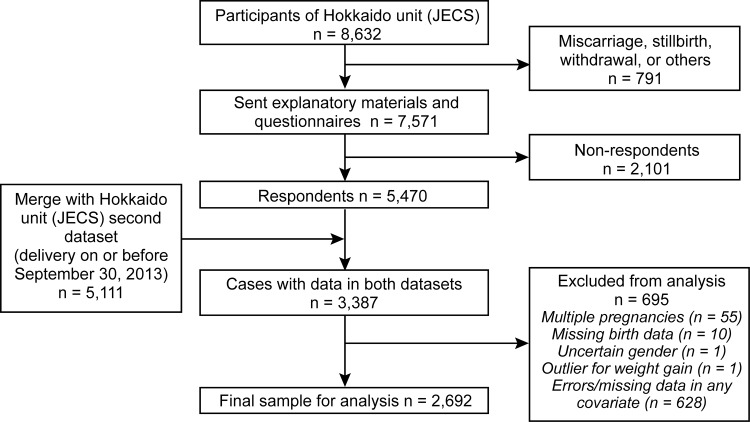
The sample selection flow diagram.

The sample selection flow diagram is shown in (Fig 1). Of the possible 8,362 study participants, we sent the self-questionnaire to 7,571 participants after their delivery. The 791 participants who were not sent questionnaires were excluded due to miscarriages, stillbirths, participation withdrawal, or for other reasons. A total of 5,468 agreed to participate in the study, indicating a response rate of 72.2%.

The questionnaire data on yoga were then merged with a second dataset provided by the JECS Hokkaido unit. This second dataset included participants who gave birth on or before September 30, 2013, and was released in June 2015. After merging the data, the sample included 3,387 women who had corresponding data in both datasets. We excluded those with multiple pregnancies (n = 55), missing birth data (n = 10), and uncertain gender (n = 1). An outlier who experienced weight gain of 30 kg or more during pregnancy (n = 1) and participants with errors/missing data in any of the covariates (n = 628) were also excluded. The final study sample for analysis comprised 2,692 women.

### Ethical Issues

This adjunct study of the JECS was approved by the Institutional Ethics Boards for Epidemiological Studies at Asahikawa Medical University, the Hokkaido University Center for Environment and Health Sciences, and the Japan Red Cross Hokkaido College of Nursing.

### Exposure Variable

The exposure variable analyzed in this study was the practice of prenatal yoga during the index pregnancy period. A self-questionnaire was sent to each participant after delivery. The median period between delivery and response to the questionnaire was approximately 3 months (mean, 2.7 months; range, 0–26). The main question in the self-questionnaire was “Did you practice prenatal yoga during this pregnancy period?” If the answer was “Yes”, the participant was categorized into the “prenatal yoga group”; if the answer was “No” or “I tried to, but was stopped by the physician”, the participant was categorized into the “non-prenatal yoga group”. Respondents in the prenatal yoga group were then directed to answer additional questions that addressed practice methods (use of instructor or self-study by DVDs or books), practice frequency (duration of practice in gestational weeks, frequency of practice per week, and duration of practice in minutes per exercise session), and the specific components of yoga (inclusion or non-inclusion of physical posture, breathing techniques, and meditation practice).

### Outcome Variable

The outcome variable analyzed in this study was the use of ritodrine hydrochloride. The use of ritodrine hydrochloride once or more during the index pregnancy period was ascertained from medical records by physicians, midwives, or JECS research coordinators after delivery.

### Covariates

We collected information on various potential confounding factors of preterm delivery [29–31] and prenatal yoga. Using a self-questionnaire sent to the participants between 12 and 16 weeks of gestation, we collected data on marital status, employment status, physical activity level before pregnancy (metabolic equivalents [METS]×min/day) [32,33], and malformation of uterus, if any.

Using another self-questionnaire sent to the participants between 22 and 28 weeks of gestation, we collected data on smoking, alcohol consumption, and maternal education.

In the post-delivery self-questionnaire that addressed prenatal yoga, we additionally collected data on whether each participant had intended to practice prenatal yoga at approximately 15 weeks of gestation and whether they had undertaken any complementary therapies during pregnancy. These factors were analyzed due to the assumption that people with interest in prenatal yoga may have higher health consciousness and engage in other activities that they perceive to be healthy.

The following information were also collected from medical records: maternal age at delivery, prenatal care hospital, parity, pre-pregnancy body mass index (BMI), infertility treatment, history of preterm delivery, history of spontaneous abortion, chronic hypertension, diabetes mellitus, psychiatric illness, hypothyroidism, autoimmune disease, gender of offspring, use of iron preparations during pregnancy, and threatened abortion during pregnancy. In addition, hospitals that offered in-hospital prenatal yoga classes during the study period were identified either through their websites or phone calls.

### Statistical Analysis

Analyses were performed using IBM SPSS Statistics 23.0 for Windows (SPSS Inc., Chicago, IL, USA). Baseline characteristics were calculated and presented as numbers and percentages or means and standard deviations, where applicable. Chi-square tests or Fisher’s exact tests were performed to assess differences in baseline characteristics for categorical variables and the Mann-Whitney *U* test was used for continuous variables.

Odds ratios (ORs) with 95% confidence intervals (CIs) for ritodrine hydrochloride use according to the practice of prenatal yoga were estimated using logistic regression models. We developed 3 multivariate logistic regression models for conducting adjustments using the forced entry method. Model 1 adjusted for maternal age at delivery, participation area, parity, marital status, smoking, alcohol consumption, maternal education, pre-pregnancy BMI, employment status, physical activity level before pregnancy, intention to practice prenatal yoga at around 15 weeks of gestation, and prenatal yoga classes held at the prenatal care hospital as the baseline covariates. Model 2 adjusted for the same variables in Model 1, as well as infertility treatment, history of preterm delivery, history of spontaneous abortion, malformation of uterus, chronic hypertension, diabetes mellitus, psychiatric illness, hypothyroidism, and autoimmune disease as covariates of complications or medical history. Model 3 adjusted for the same variables in Model 2, as well as practice of alternative therapies other than prenatal yoga, gender of offspring, use of iron preparations during pregnancy, and threatened abortion during pregnancy as covariates of information obtained during pregnancy.

In order to further explore the exposure-response relationship, we analyzed the prenatal yoga group based on the following 3 practice frequency factors: number of practice weeks, number of practice sessions, and total practice duration in minutes. Participants in the prenatal yoga group were allocated into 2 groups for each of these factors based on their respective median values. Next, the OR for each of these dichotomous variables was then calculated using a logistic regression model that included the covariates described in Model 3.

We also divided the prenatal yoga group into 4 subgroups based on the combination of the start point of yoga in gestational weeks (divided according to the median value in gestational weeks) and total practice duration (divided according to the median value in minutes) to explore the following 2 possibilities: the first possibility is that participants who had started prenatal yoga earlier may demonstrate the net effect of this practice because they could be interpreted as having physically similar states to the non-prenatal yoga group with regard to experiencing uterine contractions (due to statistical adjustment of threatened abortion in the multivariate analysis). The second possibility is that participants who had started prenatal yoga later were in better condition to practice yoga due to a lack of uterine contractions.

Furthermore, we conducted sensitivity analyses using restricted samples comprising 2,542 cases (excluding 150 threatened abortion cases) and 2,632 cases (excluding 60 cases who had attempted to do prenatal yoga but were stopped by their physician). Finally, as an exploratory subanalysis on practice methods and the components of yoga, we divided the prenatal yoga group into 4 subgroups based on combinations of 3 patterns: categorization according to practice methods (instructor or self-study) and the median of total practice duration in minutes, categorization according to breathing technique use and the median of total practice duration in minutes, and categorization according to meditation use and the median of total practice duration in minutes. Statistical significance was set at P < 0.05 for all analyses.

## Results

A total of 567 (21.1%) women in the study sample reported taking part in prenatal yoga during their pregnancy period. The comparison of the baseline characteristics between the prenatal yoga group and the non-prenatal yoga group is shown in Table 1.

**Table 1:** Baseline characteristics of study participants according to practice and non-practice of prenatal yoga.

	No Prenatal Yoga (n = 2125)	Prenatal Yoga (n = 567)	
Variable	N (%)	N (%)	P value
Maternal age at delivery (y)			0.248^d^
≤24	137 (6.4)	24 (4.2)	
25–29	569 (26.8)	160 (28.2)	
30–34	800 (37.6)	213 (37.6)	
≥35	619 (29.1)	170 (30.0)	
Participation area			0.927^d^
Sapporo	1155 (54.4)	312 (55.0)	
Asahikawa	496 (23.3)	128 (22.6)	
Kitami	474 (22.3)	127 (22.4)	
Parity			<0.001^d^
0	819 (38.5)	375 (66.1)	
1	940 (44.2)	148 (26.1)	
2	309 (14.5)	36 (6.3)	
≥3	57 (2.7)	8 (1.4)	
Marital status			0.119^d^
Married	2039 (96.0)	552 (97.4)	
Single (Unmarried, divorced, or widowed)	86 (4.0)	15 (2.6)	
Smoking at the second trimester			<0.001^d^
Never smoked	1108 (52.1)	351 (61.9)	
Ex-smokers who quit before pregnancy	623 (29.3)	155 (27.3)	
Ex-smokers who quit after pregnancy	302 (14.2)	56 (9.9)	
Current smokers	92 (4.3)	5 (0.9)	
Alcohol consumption at the second trimester			<0.001^d^
Never drank	568 (26.7)	107 (18.9)	
Ex-drinkers who quit before pregnancy	343 (16.1)	91 (16.0)	
Ex-drinkers who quit after pregnancy	1134 (53.4)	357 (63.0)	
Current drinkers	80 (3.8)	12 (2.1)	
Maternal education			<0.001^d^
Junior high school	83 (3.9)	15 (2.6)	
High school	674 (31.7)	112 (19.8)	
Junior college, vocational school, or specialized vocational high school	935 (44.0)	259 (45.7)	
University or graduate school	433 (20.4)	181 (31.9)	
Pre-pregnancy BMI (kg/m2)			<0.001^d^
<18.5	352 (16.6)	108 (19.0)	
18.5–24.9	1522 (71.6)	427 (75.3)	
≥25	251 (11.8)	32 (5.6)	
Employment status			0.027^d^
Housewife	916 (43.1)	217 (38.3)	
Regular employee or self-employed	687 (32.3)	206 (36.3)	
Temporary staff, part-time staff, or commissioned staff	447 (21.0)	113 (19.9)	
Unemployed or others	75 (3.5)	31 (5.5)	
Physical activity level before pregnancy (IPAQ)			
METS × min/day (Mean [SD])	403.1 (705.0)	374.5 (636.7)	0.396^f^
Intention to do prenatal yoga at around 15 weeks of gestation	1395 (65.6)	539 (95.1)	<0.001^d^
Prenatal yoga classes were held in prenatal care hospital	1458 (68.6)	437(77.1)	<0.001^d^
Infertility treatment			0.131^d^
None (Spontaneous pregnancy)	1986 (93.5)	521 (91.9)	
Ovulation induction or AIH	100 (4.7)	38 (6.7)	
ART	39 (1.8)	8 (1.4)	
History of preterm delivery^a^	57 (4.4)	7 (3.6)	0.646^d^
History of spontaneous abortion^b^	417 (27.1)	101 (36.3)	0.002^d^
Malformation of uterus	10 (0.5)	1 (0.2)	0.476^e^
Chronic hypertension	38 (1.8)	7 (1.2)	0.361^d^
Diabetes mellitus	29 (1.4)	5 (0.9)	0.360^d^
Psychiatric illness	8 (0.4)	1 (0.2)	0.694^e^
Hypothyroidism	16 (0.8)	12 (2.1)	0.004^d^
Autoimmune disease	10 (0.5)	2 (0.4)	1.000^e^
Practice of alternative therapies other than prenatal yoga^c^	383 (18.0)	195 (34.4)	<0.001^d^
Gender of offspring (Male)	1121 (52.8)	292 (51.5)	0.595^d^
Use of iron preparations during pregnancy	611 (28.8)	178 (31.4)	0.220^d^
Threatened abortion during pregnancy	128 (6.0)	22 (3.9)	0.048^d^
Use of ritodrine hydrochloride	651 (30.6)	139 (24.5)	0.004^d^

^a^ Only among women with parity ≥1 (n = 1,498).

^b^ Only among women with gravida ≥1 (n = 1,815).

^c^ Includes Lamaze technique, sophrology, aromatherapy, maternity swimming, maternal aerobics, massage, acupuncture, Qigong, Tai Chi, meditation, hypnotherapy, or autogenic training.

^d^ χ2 test.

^e^ Fisher's exact test.

^f^ Mann-Whitney U test.

BMI, Body Mass Index; SD, Standard Deviation; IPAQ, International Physical Activity Questionnaire; AIH, Artificial insemination with husband's sperm; ART, Assisted reproduction technology

After adjusting for covariates (Table 2), the practice of prenatal yoga had a significantly lower OR for the use of ritodrine hydrochloride (adjusted OR, 0.77; 95% CI, 0.61–0.98).

**Table 2:** Adjusted odd ratios for ritodrine hydrochloride use.

	OR	95%CI	P value
No prenatal yoga	1 (Ref)		
Prenatal yoga (Crude)	0.74	(0.60–0.91)	0.005
(Model 1)	0.77	(0.61–0.97)	0.030
(Model 2)	0.78	(0.62–0.99)	0.041
(Model 3)	0.77	(0.61–0.98)	0.034

Model 1 adjusted for maternal age at delivery, participation area, parity, marital status, smoking, alcohol consumption, maternal education, pre-pregnancy BMI, employment status, physical activity level before pregnancy, intention to do prenatal yoga at around 15 weeks of gestation, and prenatal yoga classes held at the prenatal care hospital.

Model 2 adjusted for the variables in Model 1, as well as infertility treatment, history of preterm delivery, history of spontaneous abortion, malformation of uterus, chronic hypertension, diabetes mellitus, psychiatric illness, hypothyroidism, and autoimmune disease.

Model 3 adjusted for the variables in Model 2, as well as practice of alternative therapies other than prenatal yoga, gender of offspring, use of iron preparations during pregnancy, and threatened abortion during pregnancy.

OR, odds ratio; CI, confidence intervals

In an analysis of practice frequency types (Table 3), the ORs for ritodrine hydrochloride use were significantly lower for higher numbers of practice weeks (adjusted OR, 0.67; 95% CI, 0.48–0.93), higher numbers of practice sessions (adjusted OR, 0.62; 95% CI, 0.44–0.86), and higher total practice duration in minutes (adjusted OR, 0.54; 95% CI, 0.38–0.76) relative to the non-prenatal yoga group.

**Table 3:** Adjusted odd ratios for ritodrine hydrochloride use stratified by the number of yoga practice weeks, number of practice sessions, and total practice duration in minutes.

	OR	95%CI	P value	P value for trend
**Number of practice weeks**				
No prenatal yoga (n = 2125)	1 (Ref)			0.016
Prenatal yoga ≤13 weeks (n = 294)	0.86	(0.63–1.17)	0.332	
Prenatal yoga >13 weeks (n = 251)	0.67	(0.48–0.93)	0.019	
**Number of practice sessions**				
No prenatal yoga (n = 2125)	1 (Ref)			0.007
Prenatal yoga ≤17 sessions (n = 279)	0.92	(0.67–1.25)	0.581	
Prenatal yoga >17 session (n = 264)	0.62	(0.44–0.86)	0.005	
**Total practice duration in minutes**				
No prenatal yoga (n = 2125)	1 (Ref)			0.002
Prenatal yoga ≤900 min (n = 269)	1.04	(0.76–1.42)	0.804	
Prenatal yoga >900 min (n = 267)	0.54	(0.38–0.76)	<0.001	

Adjusted for maternal age at delivery, participation area, parity, marital status, smoking, alcohol consumption, maternal education, pre-pregnancy BMI, employment status, physical activity level before pregnancy, intention to do prenatal yoga at around 15 weeks of gestation, prenatal yoga classes held at the prenatal care hospital, infertility treatment, history of preterm delivery, history of spontaneous abortion, malformation of uterus, chronic hypertension, diabetes mellitus, psychiatric illness, hypothyroidism, autoimmune disease, practice of alternative therapies other than prenatal yoga, gender of offspring, use of iron preparations during pregnancy, and threatened abortion during pregnancy.

OR, odds ratio; CI, confidence intervals

As shown in Table 4, there was a marginally significantly lower risk of ritodrine hydrochloride use in the group that started prenatal yoga at 21 weeks of gestation or earlier and had a total practice duration of more than 900 minutes (adjusted OR, 0.69; 95% CI, 0.46–1.03). The group that started prenatal yoga at 22 weeks or later and had a total practice duration time of 900 minutes or less showed no significant relationship with ritodrine hydrochloride use (adjusted OR, 0.94; 95% CI, 0.64–1.38); in contrast, the group that started prenatal yoga at 22 weeks or later and had a total practice duration of more than 900 minutes showed a significantly lower risk for ritodrine hydrochloride use (adjusted OR, 0.34; 95% CI, 0.19–0.62).

**Table 4:** Adjusted odd ratios for ritodrine hydrochloride use stratified by the start of yoga in gestational weeks and total practice duration in minutes.

	OR	95%CI	P value	P value for trend
**Start of yoga in gestational weeks and total practice duration in minutes**				
No prenatal yoga (n = 2125)	1 (Ref)			0.001
Prenatal yoga ≤21 weeks and ≤900 min (n = 102)	1.21	(0.76–1.92)	0.413	
Prenatal yoga ≥22 weeks and ≤900 min (n = 167)	0.94	(0.64–1.39)	0.770	
Prenatal yoga ≤21 weeks and >900 min (n = 167)	0.69	(0.46–1.03)	0.069	
Prenatal yoga ≥22 weeks and >900 min (n = 100)	0.34	(0.19–0.62)	<0.001	

Adjusted for maternal age at delivery, participation area, parity, marital status, smoking, alcohol consumption, maternal education, pre-pregnancy BMI, employment status, physical activity level before pregnancy, intention to do prenatal yoga at around 15 weeks of gestation, prenatal yoga classes held at the prenatal care hospital, infertility treatment, history of preterm delivery, history of spontaneous abortion, malformation of uterus, chronic hypertension, diabetes mellitus, psychiatric illness, hypothyroidism, autoimmune disease, practice of alternative therapies other than prenatal yoga, gender of offspring, use of iron preparations during pregnancy, and threatened abortion during pregnancy.

OR, odds ratio; CI, confidence intervals

The sensitivity analysis that excluded cases with threatened abortion during pregnancy produced results that were similar to those of the main analysis (S1 Table). The other sensitivity analysis (which excluded cases who tried to do prenatal yoga but were stopped by their physician) did not show any significant relationship between prenatal yoga and ritodrine hydrochloride use (S2 Table). However, the subanalysis stratified by both the start point of prenatal yoga and total practice duration indicated a significant exposure-response relationship between prenatal yoga and ritodrine hydrochloride use, which was similar to the findings of the main analysis. The exploratory analysis of practice methods, breathing technique, and meditation (S3 Table) showed few differences in ritodrine hydrochloride use among the different practice methods and the use or non-use of meditation. Practice methods, the median values for practice frequency, and components of prenatal yoga are shown in (S4 Table).

## Discussion

After adjusting for a diverse range of covariates, the practice of prenatal yoga was found to be significantly associated with reduced ritodrine hydrochloride administration in pregnant women. Furthermore, the stratified analysis showed that a total practice duration of more than 900 minutes had a significantly protective OR with respect to ritodrine hydrochloride use. The group that started prenatal yoga at 21 weeks of gestation or earlier and had a total practice duration of more than 900 minutes also showed a marginally significant relationship with reduced ritodrine hydrochloride use.

To the best of our knowledge, there has yet to be a study that reported the effects of prenatal yoga on the use of tocolytic drugs. However, 3 interventional studies have previously analyzed the effects of prenatal yoga on prematurity[7,10,34], and found that subjects who practiced yoga had significantly lower incidences of preterm births and longer gestational periods when compared with subjects who had not practiced yoga. One of these studies was not an RCT [34] and the study subjects were selected for interventions based on the distance of their residence to the hospital, which may have led to the introduction of selection bias. As a result, the differences in the effects of prenatal yoga between that study and our findings could not be directly compared. Of the remaining 2 studies, one was an RCT conducted in the US that focused on pregnant women who also had prenatal depression [10], and the other was an RCT conducted in India that investigated pregnant women with high-risk factors that included obesity and advanced age [7]. These characteristics may limit the generalizability of those findings. Although this analysis was conducted as an observational study, the study subjects were pregnant women from the general population in Japan, thereby providing a relatively higher level of generalizability to our results. In contrast to the previous studies that focused on prematurity, the outcome measure of our study was ritodrine hydrochloride use, which may be indicative of a pregnant woman’s experience of any uterine contractions[22]. On the other hand, a previous observational study [35] showed no significant association between yoga and preterm birth, and a non-RCT study [36] was similarly unable to detect an association between yoga and the number of gestational weeks before delivery. While the results are not conclusive, this study conducted a multifaceted analysis that adjusted for many covariates, and our findings indicate that prenatal yoga has possible preventive effects on ritodrine hydrochloride use.

Although the mechanism underlying the reduction of ritodrine hydrochloride use through prenatal yoga remains unclear, uterine contractions induced by the inflammatory process may be a possible cause. Bacterial infection has been documented to be one of the main causes of spontaneous preterm delivery [37–39]. Furthermore, the roles for the pro-inflammatory cytokines interleukin (IL)-1β, IL-6, IL-8, and tumor necrosis factor alpha (TNF-α) are evident in both full-term and preterm delivery, and have been shown to be independent of the presence of infections [38,40,41]. This indicates that the inflammatory process, regardless of bacterial infections, may result in uterine contractions that lead to preterm labor. The beneficial role of yoga has also been demonstrated in non-pregnant women, where an RCT study of breast cancer survivors showed a significant dose-response decrease in serum IL-1β and IL-6 in those who practiced yoga [3]. It is possible that a reduction in the inflammatory response in the prenatal yoga group was able to suppress uterine contractions and reduce the need for ritodrine hydrochloride prescriptions.

The strengths of this study are 1) a substantially large sample size, 2) demonstration of the existence of an exposure-response relationship between prenatal yoga and ritodrine hydrochloride use, 3) the examination of medical records to ascertain ritodrine hydrochloride use and numerous covariates, 4) statistical adjustment of many potential confounders, 5) provision of relatively detailed insight into the benefits of prenatal yoga, and 6) production of comparatively generalizable results for pregnant Japanese women.

This study has several limitations. First, our results include the possibility of reverse causality, where participants in the prenatal yoga group may have been able to exercise only because they were not impeded by uterine contractions. In fact, the OR for ritodrine hydrochloride use was lower in the group that started prenatal yoga at 22 weeks or later and had a total practice duration of more than 900 minutes when compared with the group that started prenatal yoga at 21 weeks of gestation or earlier. Additionally, our sensitivity analyses showed the absence of a significant relationship between ritodrine use and prenatal yoga. It is therefore plausible that these results may reflect the presence of reverse causality. However, we demonstrated that the group that started prenatal yoga at 21 weeks of gestation or earlier and had a total practice duration of more than 900 minutes had a marginally significant lower risk for ritodrine hydrochloride use after adjusting for many covariates (including threatened abortion), and the sensitivity analysis models also indicated a significant exposure-response relationship. Additionally, as shown in Table 3, the ORs among high frequency groups were lower in the “Total practice duration in minutes” group (0.54) than the “Number of practice sessions” group (0.62) or the “Number of practice weeks” group (0.67). This suggests that the longer a participant actually practiced yoga, the lower the OR of being administered ritodrine; these observations corroborate the presence of an exposure-response relationship. Second, the information on prenatal yoga was collected retrospectively after delivery (mean: 2.7 months post-delivery), and the reliability and validity of the original questionnaire were not examined. There may have been some pregnant women who practiced prenatal yoga up until delivery, and information acquisition should therefore have ideally been conducted at the time of admission for delivery. Despite this limitation, we were able to carry out the survey at a relatively close time frame after delivery. Third, some participants in the prenatal yoga group may have practiced yoga after having received ritodrine hydrochloride, which may have led to an underestimation of the potential benefits of yoga and prevented the observation of statistically significant associations in several aspects of the analyses. Fourth, ritodrine hydrochloride can be administered either orally or through infusions. Approximately 14.1% (380/2,692) of study participants had been diagnosed with preterm labor, whereas approximately 29.3% (790/2,692) were administered ritodrine. In this regard, it is possible that ritodrine was administered via the oral route to women without a diagnosis of preterm labor but had experienced relatively mild uterine contractions. In other words, approximately half of the ritodrine-administered participants may have reported experiencing mild uterine contractions. Despite the differences in administration routes, both these methods are likely to reflect the participants’ experience of uterine contractions [22], which may be an indicator of preterm labor. Fifth, the use of ritodrine hydrochloride is dependent on the discretion of each obstetrician, which may have introduced a degree of bias into the study. However, this analysis adjusted for variations in participation regions and for prenatal yoga classes that were held in the prenatal care hospital. Sixth, the number of excluded participants from this analysis was 695 from the original 3387 (20.5%) patients, and the majority of these cases were excluded due to missing values. However, a supplementary analysis showed that the proportions of patients who practiced prenatal yoga and were administered ritodrine hydrochloride did not differ between these excluded subjects and subjects who were included in the final analysis (data not shown). Seventh, there is the possibility that residual confounding factors remain unmeasured. Our study adjusted for numerous variables (including smoking status and pre-pregnancy BMI), many of which were assessed from medical records. However, we did not adjust for psychological variables, which may have influenced our results. Eighth, the outcome in this analysis was ritodrine hydrochloride use, and we could not directly analyze preterm birth or threatened preterm labor due to low statistical power. Consequently, we are unable to make any conclusions on the direct relationship between prenatal yoga and preterm birth or threatened preterm labor. However, we intend to analyze these outcomes in the future with a larger sample size after improvements have been made to the JECS data.

Prenatal yoga was associated with a lower risk of ritodrine hydrochloride use in pregnant Japanese women, especially those with a total practice duration that exceeded 900 minutes. This result suggests that performing prenatal yoga for a cumulative total of more than 900 minutes during pregnancy may counter the onset of uterine contractions that necessitates the use of ritodrine hydrochloride. Prenatal yoga may therefore be a viable and beneficial option for pregnant women in the selection of alternative therapies.

## Supporting Information

S1 Table Adjusted odd ratios for ritodrine hydrochloride use among women without threatened abortion. (XLSX)

S2 Table Adjusted odd ratios for ritodrine hydrochloride use among women with the exclusion of those who attempted to start prenatal yoga but were stopped by their physician. (XLSX)

S3 Table Adjusted odd ratios for ritodrine hydrochloride use stratified by practice methods, breathing technique use, meditation use, and total practice duration in minutes. (XLSX)

S4 Table Practice methods, practice frequency, and the components of prenatal yoga. (XLSX)
